# Supplementary material for: Targeting SAM-I Riboswitch Using Antisense Oligonucleotide Technology for Inhibiting the Growth of Staphylococcus aureus and Listeria monocytogenes
Source: Antibiotics (Basel). 2022 Nov 19;11(11):1662. doi: 10.3390/antibiotics11111662 (PMC9686682; doi:10.3390/antibiotics11111662)
Supplement: Supplementary file 1 [file antibiotics-11-01662-s001.zip › antibiotics-1960631-supplementary.pdf]

Supplementary data

Targeting SAM-I riboswitch using antisense oligonucleotide technology for inhibiting the growth of Staphylococcus aureus and Listeria monocytogenes

Martina Traykovska and Robert Penchovsky*

Department of Genetics, Faculty of Biology, Sofia University “St. Kliment Ohridski”

Sofia, Bulgaria

*To whom the correspondence should be addressed:

Department of Genetics,

Faculty of Biology,

Sofia University “St. Kliment Ohridski”,

8 Dragan Tzankov Blvd.,

1 164 Sofia, Bulgaria,

Office phone: +35928167340

e-mail: robert.penchovsky@hotmail.com

Robert Penchovsky’s ORCID: https://orcid.org/0000-0001-9502-3421

Table S1. Overview of the antibiotic resistance of the human pathogenic bacteria containing the SAM-I riboswitch.

Riboswitch for SAM-I is found only in Gram-positive pathogenic bacteria. This riboswitch is present in 9 pathogenic bacteria: *Bacillus anthracis, Clostridium perfringens, Listeria monocytogenes, Staphylococcus aureus, Clostridium botulinum, Clostridium tetani, Clostridium difficile, Staphylococcus saprophyticus* and *Streptococcus epidermidis*.

Human pathogen		Presence of SAM-I	Gram - Gram +	Infectivity	Antibiotic resistance	Diseases caused by human
1	*Bacillus anthracis*	yes	+	pathogenic	closely expressed	anthrax
2	*Clostridium perfringens*	yes	+	conditionally pathogenic	closely expressed	gangrene, food poisoning
3	*Listeria monocytogenes*	yes	+	pathogenic	expressed	listeriosis, meningitis
4	*Staphylococcus aureus*	yes	+	conditionally pathogenic	widely expressed	infections

5	*Clostridium botulinum*	yes	+	pathogenic	closely expressed	botulism
6	*Clostridium tetani*	yes	+	pathogenic	expressed	tetany
7	*Clostridium difficile*	yes	+	conditionally pathogenic	widely expressed	Ab-associated diarrhea
8	*Staphylococcus saprophyticus*	yes	+	conditionally pathogenic	widely expressed	urological infections
9	*Streptococcus epidermidis*	yes	+	pathogenic	widely expressed	infections

Although the drugs mentioned above have been reported to have a lower effect due to resistant cases, they can still be used in different countries due to differences in the bacterial strains, different levels of resistance, and the perception of the local health authorities.

One of the genes controlled by this riboswitch is the yitJ gene in Bacillus subtilis. The yitJ gene is involved in the cycle of SAM synthesis. In the absence of SAM, the SAM-I mRNA is fully transcribed (Figure 1A). The primary key to triggering the transcriptional termination is the binding of SAM with the aptamer domain, stabilizing the P1 helix, causing the formation of a terminator hairpin structure stem, and dissociating the RNA polymerase of the promoter sequence (Figure 1B).

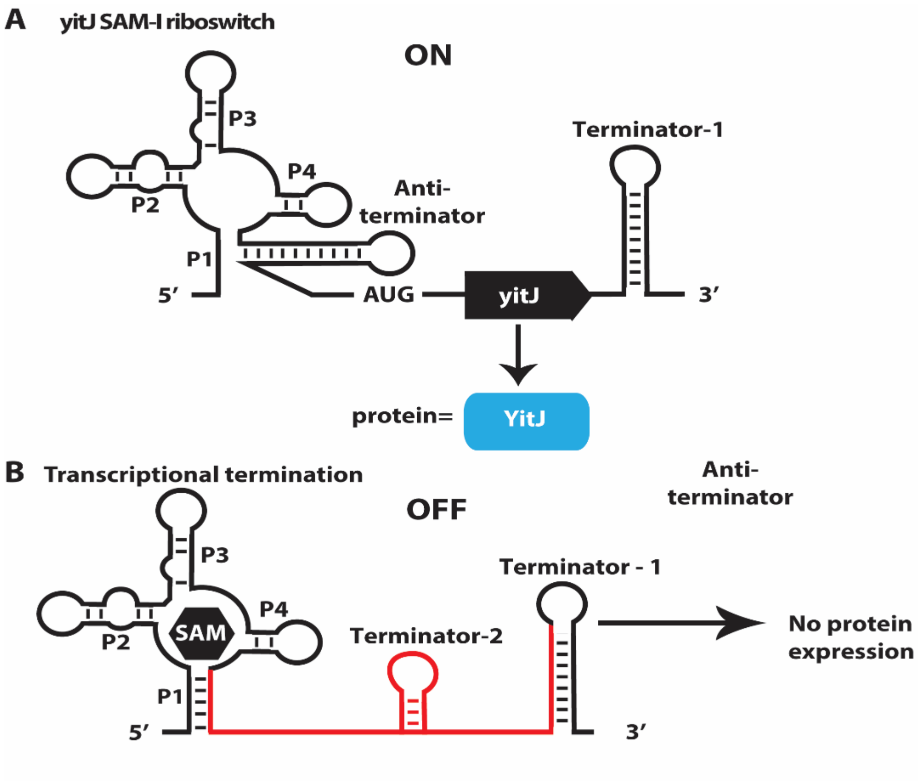

Figure S1. Regulation of gene expression through transcriptional termination by the SAM-I riboswitch. (A) In the absence of SAM, the yitJ gene is transcribed as the aptamer folds into a structure that enables the formation of an anti-terminator structure. (B) In the presence of SAM, it binds with the aptamer and folds into a structure that leads to the formation of the terminator structure. This leads to premature termination of the yitJ mRNA and no enzyme synthesis.

Biochemical pathways of SAM synthesis

Cysteine and methionine are sulfur-containing amino acids. The L-cysteine is synthesized from the serine through glycine, serine, and threonine metabolism pathway (Figure 2, reaction 1).[7c] Methionine-derived homocysteine is used as a sulfur source, and its condensation product with serine (L-cystathionine) is converted into L-cysteine, which is also involved in taurine and hypotaurine metabolism (Figure 2, reaction 2). L-cysteine is also involved in taurine and hypotaurine metabolism (Figure 2, reaction 3). Moreover, L-cysteine can be metabolized into 3-mercaptolactate and pyruvate (Figure 2, reaction 4).[7c]

Methionine is an essential amino acid that animals cannot synthesize. In bacteria, methionine is synthesized from L-aspartate, which comes from the aspartate metabolism pathway (Figure 2, reaction 5).[7d] The biosynthesis pathway of methionine from L-aspartate is as follows: L-homoserine → O-succinyl-L-homoserine (Figure 2, reaction 6) → L-cystathionine (Figure 2, reaction 7) → Homocysteine (Figure 2, reaction 8) → Methionine (Figure 2, reaction 9). Methionine might also be imported to the cell through the ABC transporter MetN (metNPQ operon) (Figure 2, reaction 10). The cobalamin-independent methionine synthase (MetE) and homocysteine S-methyltransferase (YitJ) are involved in synthesizing methionine from homocysteine. The SAM-I riboswitch controls them by transcriptional termination (Figure 2, reaction 11). The methionine salvage pathway is a universal pathway in which sulfur-containing metabolites are converted into the amino acid methionine. That pathway is found in all organisms, from unicellular bacteria to plants and animals, and starts with 5′-methylthioadenosine (MTA). Next, seven additional enzymatic steps, catalyzed by

seven proteins, form 4-methylthio-2-oxobutanoate, the deaminated form of methionine.[10]

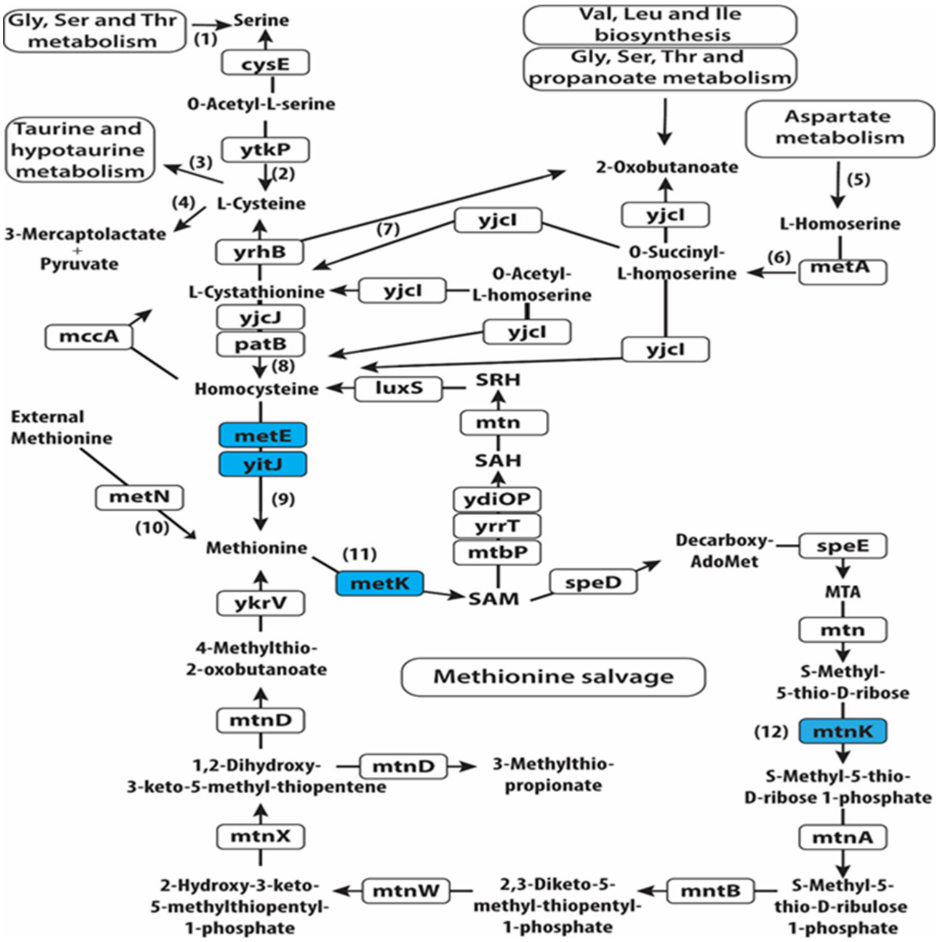

Figure S2. Biosynthetic pathway of synthesis of SAM-I riboswitch. SAM is synthesized from ATP and methionine by SAM synthetase, encoded by metK gene. SAM is also used to regenerate methionine in the methionine salvage pathway. (1) SAM can be synthesized through cysteine, and methionine is a sulfur-containing amino acid. (2) In B. subtilis 168, L-cysteine is synthesized from the serine through glycine, serine, and threonine metabolism pathway. (3) L-cysteine is synthesized through the conversion of L-cystathionine. (3) (4) L-cysteine is also involved in taurine and hypotaurine metabolism. (5) Methionine can also be synthesized from L-aspartate, which comes from the aspartate metabolism pathway. (6) Conversion of L-homoserine to O-succinyl-L-homoserine. (7) Conversion of O-succinyl-L-homoserine to L-cystathionine. (8) Conversion of L-

cystathionine to Homocysteine. (9) Conversion of Homocysteine to Methionine. (10) Methionine Import through the ABC transporter MetN (metNPQ operon). (11) The cobalamin-independent methionine synthase (MetE) and homocysteine S-methyltransferase (YitJ) are involved in the synthesis of methionine from homocysteine. They are controlled by the SAM-I riboswitch by transcription termination. (12) Synthesis of SAM by the methylthioribose kinase (MtnK), which is under the control of the SAM-I riboswitch.

One of them, the methylthioribose kinase (MtnK), is controlled by the SAM-I riboswitch. MtnK is part of the mtnKA operon (Figure 2, reaction 12). The SAM synthetase encodes the essential metK gene and is also controlled by the SAM-I riboswitch. SAM is a methyl group donor in many important transfer reactions, such as DNA methylation. SAM may also be used to regenerate methionine in the methionine salvage pathway. S-adenosyl-(L)-homocysteine (SAH) is formed by the demethylation of SAM. Furthermore, SAH is first hydrolyzed to S-ribosyl-L-homocysteine (SRH) by the MTA/SAH nucleosidase (Mtn), followed by conversion to L-homocysteine by S-ribosylhomocysteine lyase (LuxS).

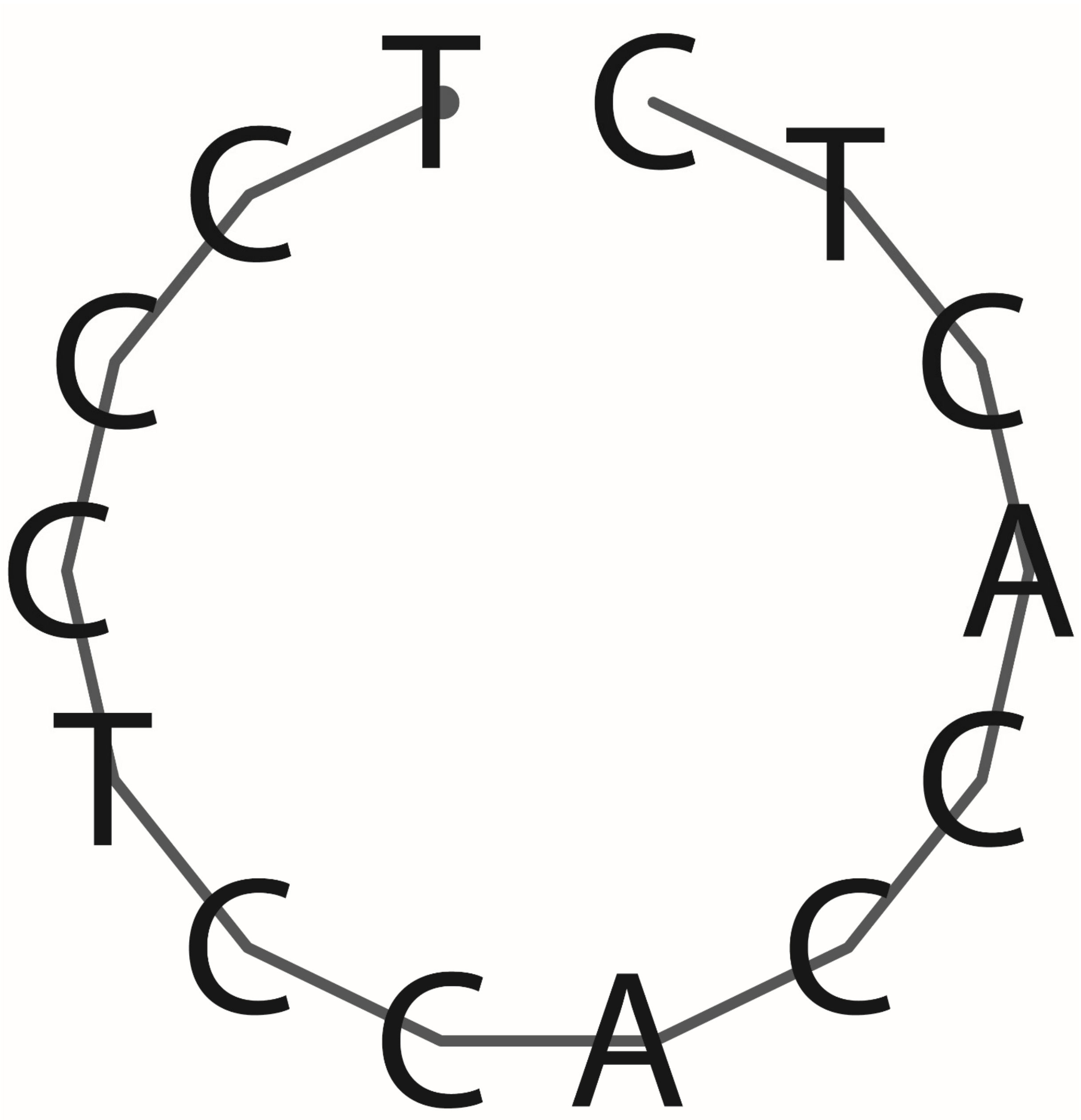

Figure S3. Lack of any secondary structures of ASO-1 as computed by the partition function of Vienna RNAfolding server.

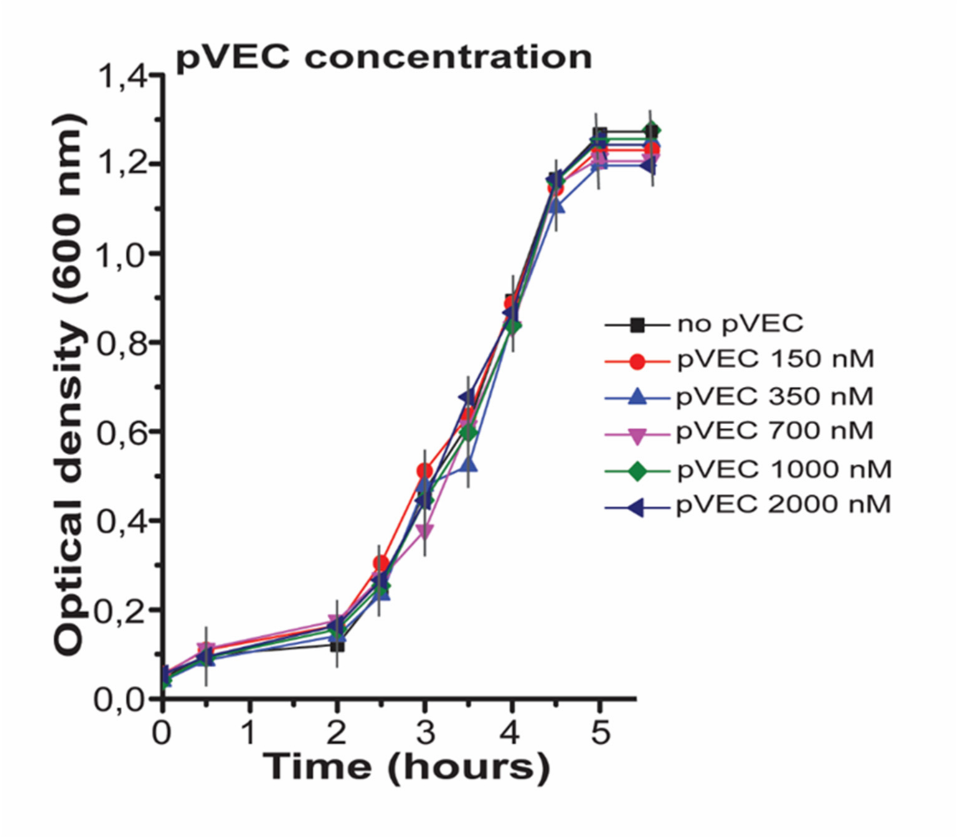

Figure S4. Testing the antibacterial activity of pVEC in all concentrations used in cells of *Staphylococcus aureus*. Lack of inhibition of the bacterial growth of Staphylococcus aureus in all tested concentrations of pVEC and without it serves as a control measurement [6,7].

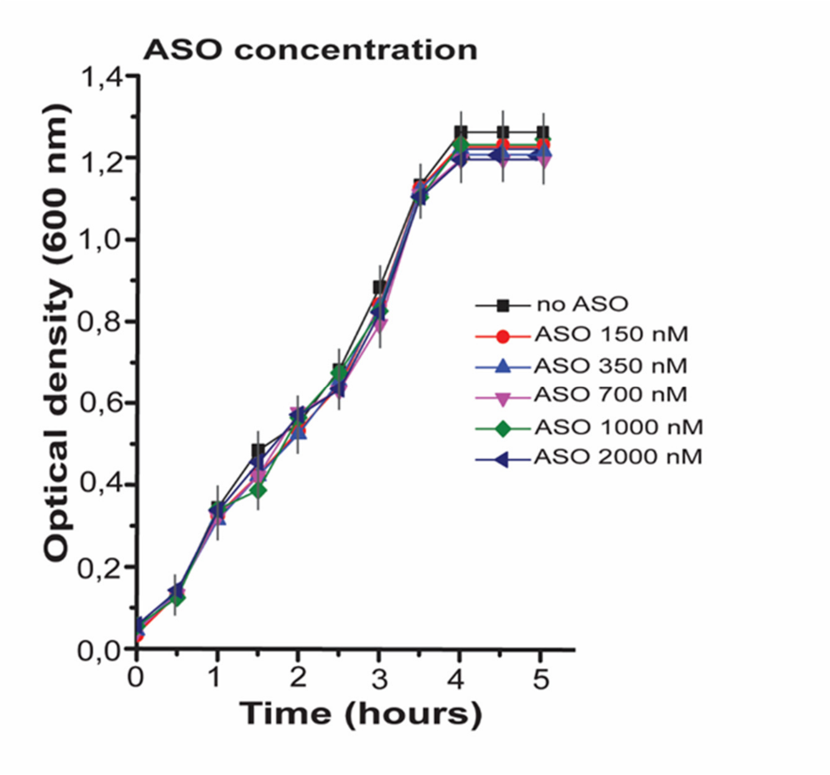

Figure S5. Testing the antibacterial activity of ASO-1 without pVEC in all concentrations in cells of *Staphylococcus aureus.* There is no inhibition of bacterial growth of Staphylococcus aureus when cells were treated with ASO-1 only. Without pVEC, ASO-1 cannot enter the bacterial cell, which serves as a control measurement [6,7].

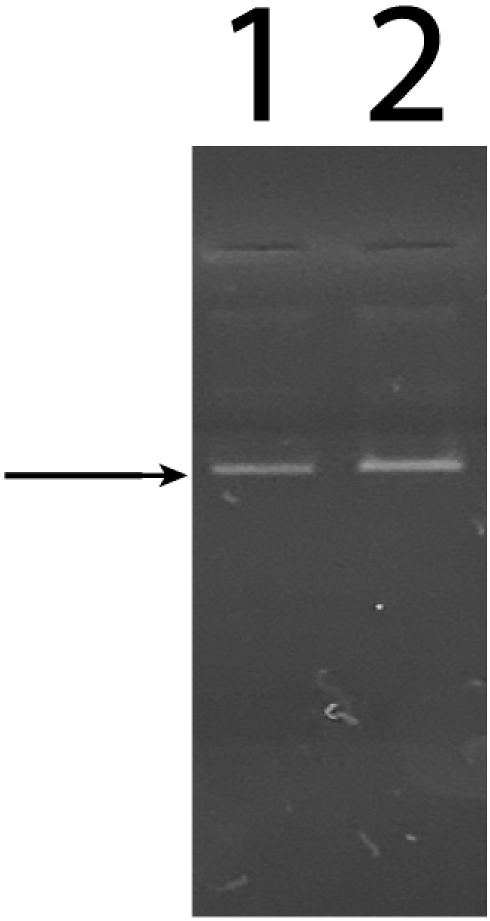

Figure S6. Testing the cDNA amplifications with primers for FMN mRNA of cDNAs treated not with pVEC-ASO-1 (lane 1) and treated with pVEC-ASO-1 (lane 2). As expected, there is no difference in the PCR amplifications of FMN mRNA between lanes 1 and 2.
